# Ipsilateral femoral neck and shaft fracture in children: Two case reports

**DOI:** 10.1097/MD.0000000000023616

**Published:** 2021-01-29

**Authors:** Hailin Xing, Quanzhou Wu, Shuhua Lan, Chong Wang, Jifei Ye, Fang Ye, Shuming Huang

**Affiliations:** Department of Orthopaedic Surgery, Lishui Hospital, Zhejiang University School of Medicine, The Fifth Affiliated Hospital of Wenzhou Medical University, Lishui Municipal Central Hospital, Lishui, Zhejiang, People's Republic of China.

**Keywords:** children, femoral shaft fracture, ipsilateral femoral neck fracture

## Abstract

**Rationale:**

Pediatric femoral shaft combined with ipsilateral femoral neck fractures are very rare but challenging injuries fraught with the development of avascular necrosis, coxa vara, and leg length discrepancy. Majority of the previous reports indicated the neck femur fracture was fixed with cannulated screws or/and pins, femoral shaft fracture was stabilized with a plate and screws. However, we used cannulated screws combined with elastic stable intramedullary nails to minimally invasive procedures treat this type of injury and achieved good follow-up results.

**Patient concerns:**

A 7-year-old boy (Case 1) was hospitalized due to a traffic accident resulting in swelling and deformity of the right thigh accompanied by limited mobility of hip and knee. A 5-year-old male child (Case 2) presented with pain and swelling in the bilateral lower limb after fall from approximately 12 feet.

**Diagnoses:**

Physical examination, X-ray film, and computed tomography were performed. Both patients were diagnosed with ipsilateral femoral neck and shaft fracture.

**Interventions:**

The fractures were reduced closed by image-intensifier imaging. Two partially threaded cancellous screws were used to fix femoral neck fracture, and elastic intramedullary nails were performed to stable the femoral shaft fracture. Postoperatively, the patients were immobilized in a one-and-a-half hip spica cast for six weeks. The internal fixations were removed after one year.

**Outcomes:**

Case one was follow-up at 14 months and the other one was followed up for 3 years. And at the last follow-up showed a normal and painless hip function. No clinical complications were found during follow-up visit, including head penetration, implant failure, fracture nonunion, avascular necrosis and hip varus deformity.

**Lessons:**

Clinician should carefully check and read relevant imaging data to avoid missed diagnosis. And the internal fixation method described in this paper may be more minimally invasive.

## 1 Introduction

Femoral shaft fractures in children are common, but concomitant ipsilateral fractures of the neck and shaft of the femur are rare with very few cases reported in the literature.^[1–3]^ These injuries are caused by high-energy impact, usually in car accidents or high-altitude fall, often combined with serious damage to other body parts, and easy to miss the diagnosis.^[2]^ Although there is still no consensus on the technical approach, reduction of the fracture remains the guiding principle in the management of such injuries, with open reduction followed by internal fixation (ORIF) being the preferred treatment. In these case reports, we describe the management of ipsilateral femoral neck and shaft fracture in children.

## 2 Case presentation

The case reports were approved by the Institutional Review Board of The Central Hospital of Lishui City, and informed consent was given by patients. Written informed consent was obtained from the patients parents for the publication of this manuscript and accompanying images.

### 2.1 Case 1

**Figure 1:**
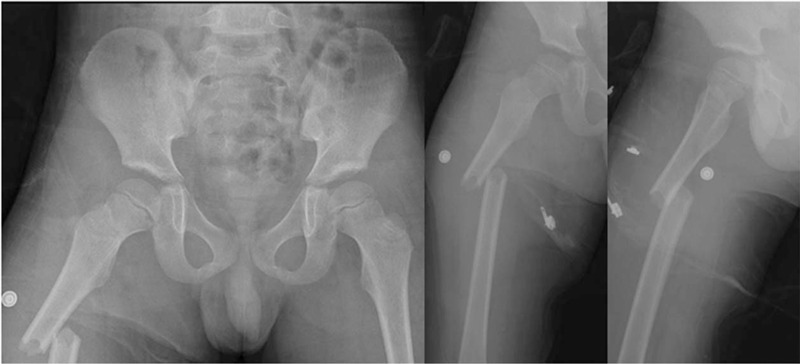
Preoperative anteroposterior and lateral x-ray showing displaced fracture of the right femoral shaft, and undisplaced femoral neck fracture.

**Figure 2:**
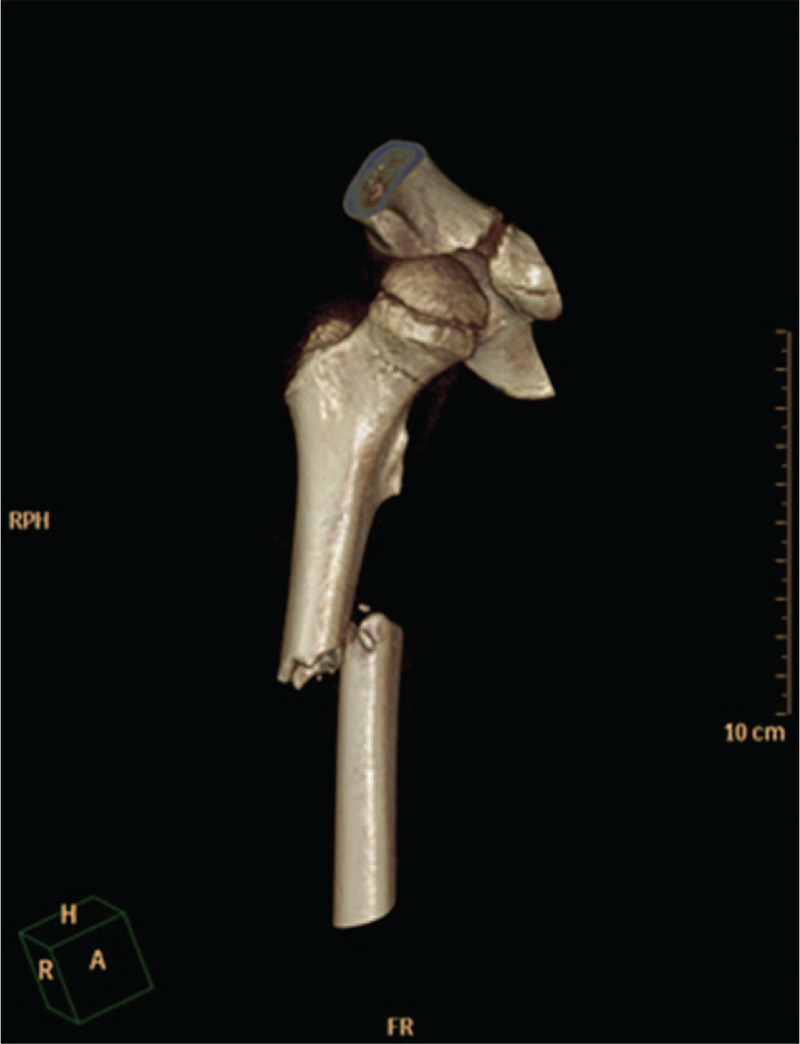
Preoperative three-dimensional CT examination of the right femur can clearly observe the femoral neck fracture line.

**Figure 3:**
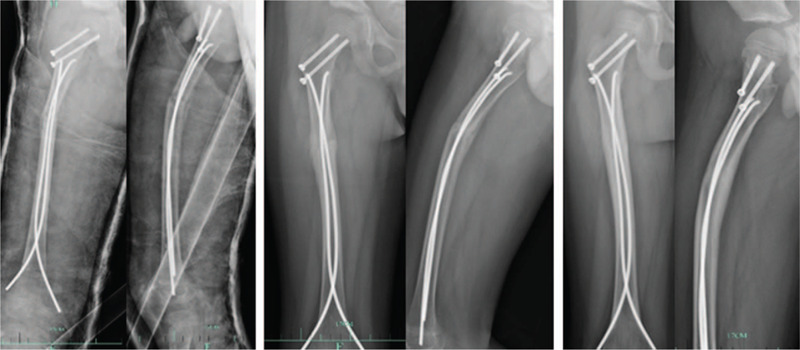
The above picture shows the X-ray findings at 1 month, 3 months, and 7 months after surgery. The osteophyte growth was observed in 1 month and the bone healing was performed in 3 months.

**Figure 4:**
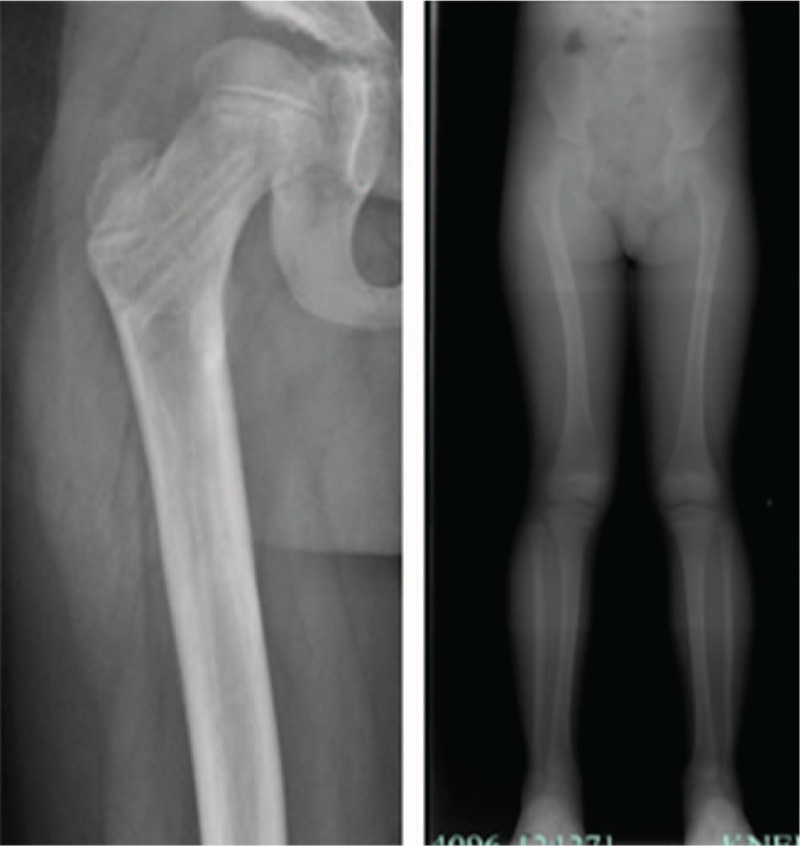
The above pictures are the right femur X-ray films after removal of internal fixation 1 year after operation, and the full length of the lower limbs after removal of internal fixation for 14 months.

**Figure 5:**
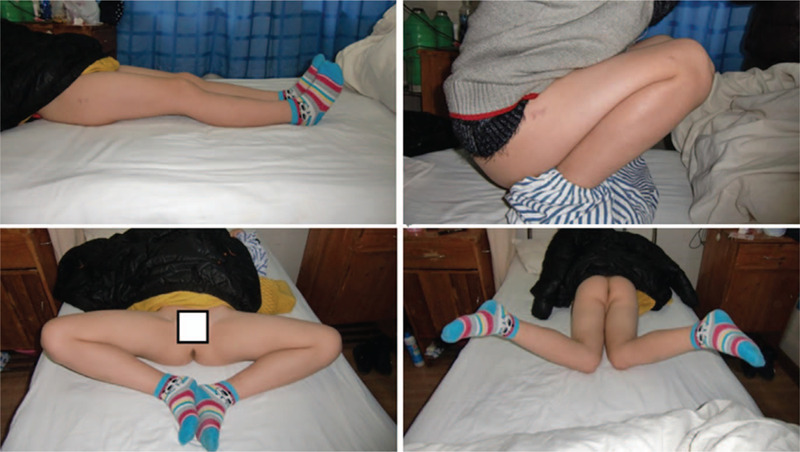
Two years after injury the patient was pain-free and regained full range of motion of the right hip and knee.

A 7-year-old boy was sent to our hospital after a traffic accident. X-ray and 3D-CT tips after admission showed right femoral neck fracture (Delbet subtype II) combined with upper one-third transverse fracture of the ipsilateral femoral shaft (AO type III), femoral shaft fracture displacement, femoral neck fracture without significant shift (Garden II type) (Figs. 1 and 2). Surgery was conducted under general anesthesia 2 days after the injury. The femoral neck was closed in supine position and 2 hollow screws (4.0 mm in diameter) were fixed. Then, the femoral shaft was closed and reset on the traction bed with 2 elastic intramedullary nails (3.0 mm) fixation (Fig. 3). After the operation, the herringbone plaster was fixed for 6 weeks. Six weeks after surgery, gypsum plaster was used, and the patient was advised to start using the lower limbs without weight bearing, hip and knee joint function exercises. Three months after the operation, crutches were used for weight walking. After the first 5 months, the patient resumed weight-bearing walking. Follow-up at 14 months after the injuries showed a normal and painless right hip function. There was no evidence of avascular necrosis, hip varus deformity, or any growth disturbance (Figs. 4 and 5).

### 2.2 Case 2

**Figure 6:**
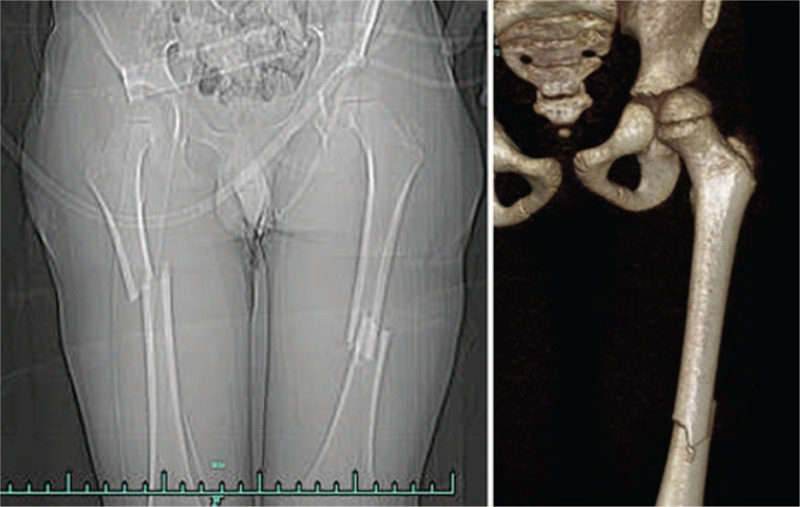
Preoperative three-dimensional CT examination of the femur can clearly observe the femoral fracture line.

**Figure 7:**
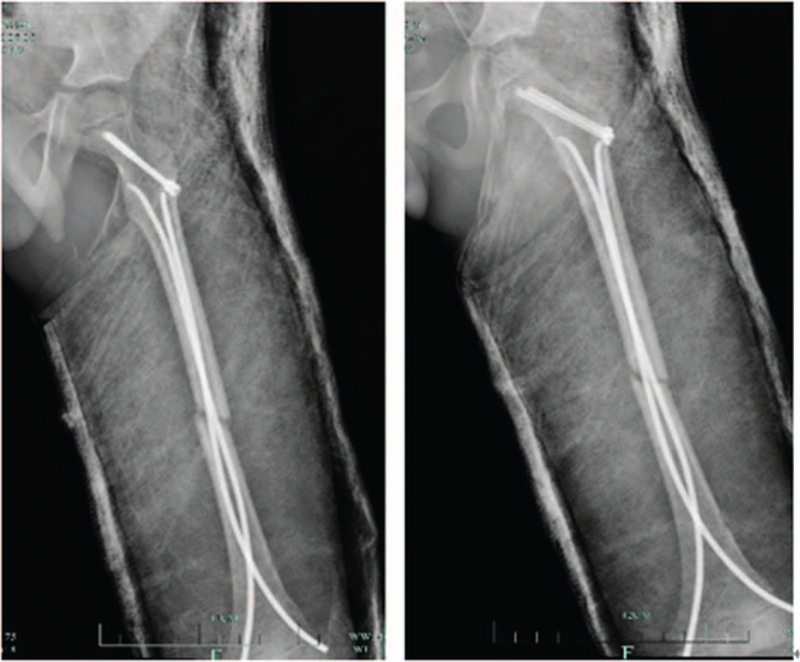
The above picture shows the X-ray findings after surgery.

**Figure 8:**
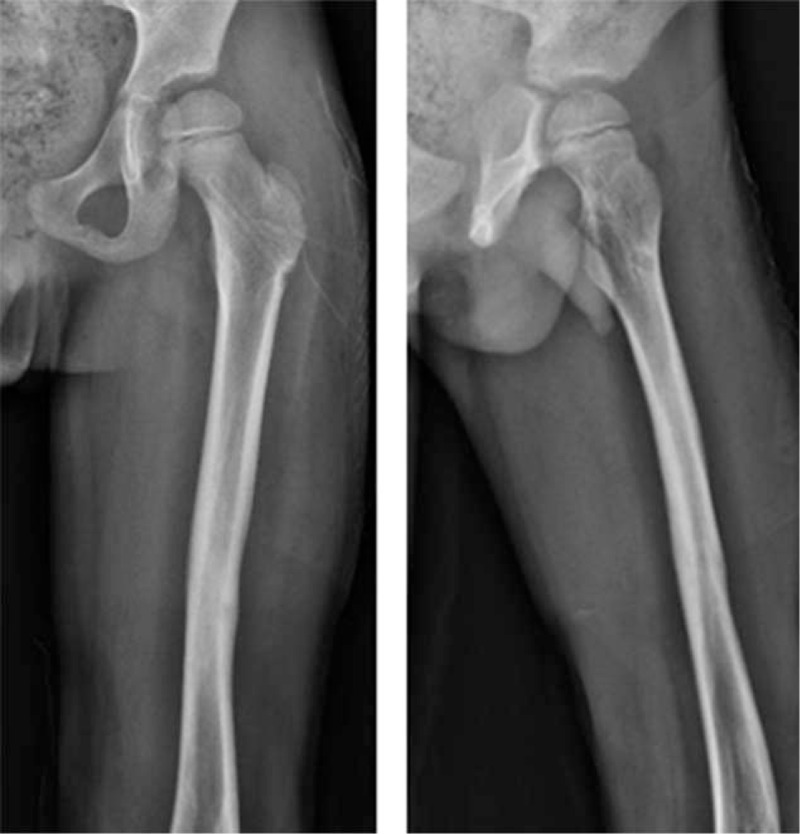
The above pictures are the left femur X-ray films after removal of internal fixation 1 year after operation.

**Figure 9:**
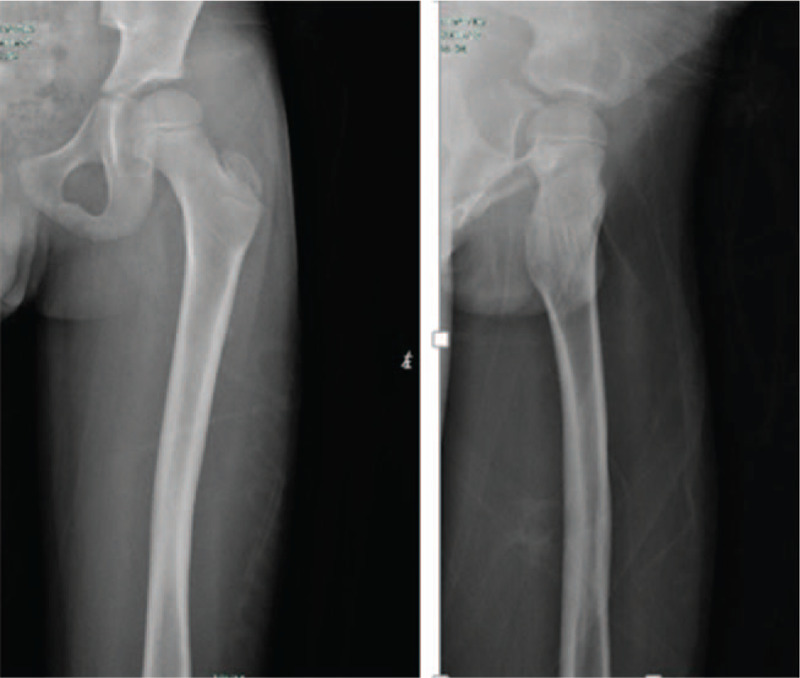
The above pictures are the left femur X-ray films after removal of internal fixation 3 year after operation.

A 5-year-old boy presented with bilateral femoral shaft fracture and left femoral neck fracture after a fall from approximately 12 feet. CT scans before surgery did not reveal signs of femoral neck fracture (Fig. 6). The right femoral shaft fracture was treated with open reduction and internal fixation. The left femoral shaft fracture was treated with closed reduction and elastic intramedullary nail fixation. During the operation, the left femoral neck fracture was found with a slight displacement. After closure and reduction, 2 4.0 mm cannulated screws were used to fix the fracture (Fig. 7). Postoperative plaster external fixation was maintained for 6 weeks. The internal fixation was removed after 1 year (Fig. 8). At the 3-year follow-up, the child had resumed normal activities and the left hip was completely painless. There was no necrosis or varus deformity in the femoral head (Fig. 9).

## 3 Discussion

Femoral shaft fractures are common in children, but femoral neck fractures are very rare, and the incidence of such combined injuries in children is only 0.7%.^[2]^ Few cases of concomitant ipsilateral fractures of the neck and shaft of the femur in children have been previously reported. McDougall first reported in 1967 a case of ipsilateral femoral neck and shaft fracture in an 8-year-old child, and since then only 16 such cases have been documented.^[2,3]^

These injuries are caused by high-energy impact, usually in car accidents or high-altitude fall, often combined with serious damage to other body parts. However, there is no unified view on the mechanism of injury. Song et al^[3]^ believed that the mechanism of injury involved high energy and impact on both the femoral shaft and neck. In contrast, Hajdu et al^[4]^ suggested that it resulted from continuous high-energy impact. When the hip is flexed and the impact is in front of the knee, the femoral shaft fracture can be caused first, and the residual impact is close to the femoral shaft.^[5,6]^ The force continues to conduct upwards, which can cause hip damage.

Among the 16 reported pediatric cases, there were 3 cases of femoral neck Delbet type I, 3 cases of type II, 8 cases of type III, 2 cases of type IV, 1 case of femoral trochanter, 7 cases of middle segment, and 3 cases of lower segment.^[2,3]^ There was 1 case of distal femoral condyle injury, 1 case of femoral shaft open fracture, and 1 case of femoral fracture. The 2 femoral neck fractures we treated were of Delbet type II, with a severe displacement of the femoral shaft fracture and no significant displacement of the femoral neck fracture. The classification of femoral neck fractures was Garden type II. By analyzing all 16 cases till date, the mechanism of fracture seems to be energy conduction attenuation, but further confirmation of biomechanics is needed.

The ipsilateral femoral neck combined with femoral shaft fractures can be treated with different approaches, including surgery and conservative treatment, or a combination of the 2 .conservative treatment can be either traction or single hip plaster fixation. Earlier reports have shown that conservative treatment can achieve better therapeutic effects. However, in the past 20 years, surgery to stabilize the fracture site has been considered as the most appropriate treatment if conditions permit, which can reduce the risk of fracture relocation, and facilitate early functional exercise for easy care.^[3]^

Ipsilateral femoral neck combined with femoral shaft fractures in adults can easily lead to missed diagnosis of hip fractures. The rate of missed diagnosis in adults can reach 11% to 33%, while the rate of missed diagnosis of hip fractures in children is higher.^[1]^ The main reason may be that such children have more complicated multiple injuries and often cannot complain of hip pain. The clinician should know the cause, symptoms and signs of the childs injury at the time of the consultation, and conduct physical examination of each affected part. X-ray imaging should include adjacent joints. For children with chest, abdomen and craniocerebral injuries, after the vital signs are stable, a comprehensive orthopedic examination should be performed. In case 2, the emergency doctor did not find a femoral neck fracture after CT. After the fixation of the femoral shaft was completed during operation, the ipsilateral femoral neck fracture was found under fluoroscopy of the C-arm machine. Although such cases may be rare, we recommend hip magnetic resonance imaging for suspected femoral neck fractures.

The femoral shaft fracture can be fixed with steel plate, elastic intramedullary nail and external fixation. However, for the ipsilateral femoral neck combined with femoral shaft fractures, external fixation stents are unsuitable. For the femoral shaft fractures in children aged 5 to 11 years, the use of elastic intramedullary nails has been widely recommended,^[7,8]^ which have the advantages of minimally invasive placement without bone damage, elastic fixation, optimal dispersion of stress, and early mobility of children after surgery. It is a common method for treating femoral shaft fractures in children.^[9]^ The risk of adverse prognosis is increased by 5 times when using elastic intramedullary nails to treat patients weighing more than 49 kg.^[10]^ For fracture types that may be axially or angularly unstable after fracture reduction, or for heavier children, we recommend using a bone plate fixation instead of an elastic intramedullary nail. However, there could be more difficulties subsequently in plate removal.

For children with femoral shaft fractures from the age of 11 years to the skeletal maturity stage, many studies recommend the use of rigid intramedullary nails with a rotor opening, bone plates and elastic intramedullary nails.^[10–12]^ It is not recommended to fix with rigid intramedullary nails through piriform or near piriform fossa^[13]^ because the subsequent treatment of patients with immature skeletal development increases the risk of avascular necrosis of the femoral head by at least 4%.^[14]^ Single intramedullary nail has been used to simultaneously fix the femoral shaft and femoral neck fractures in adults,^[15]^ but simultaneous operation of the 2 sites is difficult, even though the surgical procedure is a simple single step, as compared to the combined internal fixation, which can greatly shorten the operation time. However, some clinicians^[16,17]^ object to the use of a single intramedullary nail to fix the femoral shaft combined with femoral neck fracture because it can easily cause femoral neck nonunion or necrosis, and recommend combined internal fixation instead. At present, there is no report on the simultaneous fixation of the femoral shaft and femoral neck fractures of ipsilateral children with a single intramedullary nail.

Femoral neck fractures in children are often single plane fractures, without serrated fracture surface. The fracture surfaces are difficult to interlock with each other, and the stability is extremely poor.^[18]^ Even if there is no displacement fracture, conservative treatment can lead to secondary fracture displacement,^[19]^ so surgical reduction and internal fixation is the main method for the treatment of femoral neck fracture in children.^[18]^ It can be fixed with cannulated nails and Kirschner wire. However, the inner implants should not pass through the seesaw to avoid causing early closure. In this study, given the age of the children, the 2 femoral necks were fixed with a 4.0 mm cannulated screw and the femoral shaft was fixed with 2 3.0 mm diameter intramedullary nails.

Osteonecrosis is the most common complication of femoral neck fractures in the pediatric population because of the tenuous and changing blood supply to the femoral epiphysis. It has been proposed that osteonecrosis develops as a result of direct trauma to the vessels at the time of injury, kinking of the vessels with displacement, a tamponade effect by intracapsular hematoma, or injury during treatment. Osteonecrosis occurred after 17% to 47% of displaced fractures, but only 6% of nondisplaced fractures. Children who underwent ORIF had fewer complications as compared to those who underwent CRIF.^[18]^ One possible reason may be the release of intracapsular pressure by capsulotomy.^[20]^ Moreover, the anterior capsulotomy for ORIF does not endanger the vessels that course through the neck,^[21]^ allowing the surgeon to achieve optimal reduction, which in turn reduces complications like non-union and coxa vara.

However, Agarwal et al reported that the incidence of complications of ipsilateral femoral neck combined with femoral shaft fractures was lower than that of femoral neck fractures alone, especially femoral head necrosis. Of the 9 cases reported, 2 cases had hip varus deformity and 1 case had avascular necrosis of the femoral head. The reason may be that the impact is mostly released in the femoral shaft when the injury occurs, and the impact on the femoral neck is significantly reduced as compared to the single femoral neck fracture, resulting in a relatively low incidence of femoral head necrosis. In contrast, Song et al^[3]^ reported 5 cases, of which 2 cases had femoral head necrosis and hip varus deformity, 1 case had a simple hip varus deformity, and the higher incidence of complications was the femoral neck Delbet type I. In the present study, we preferred at least 1 attempt at closed reduction in both cases, given that an open procedure would be inappropriate for the young patients. In both cases, the femoral neck Delbet type II, femoral head necrosis, hip varus, nonunion of the fracture and early closure of the ankle did not occur. Given the small number of cases reported till date, large sample sizes and related biomechanical studies are needed in the future to understand the injury mechanisms and complications.

## 4 Conclusion

Ipsilateral femoral neck and shaft fracture in children are rare. Identifying this type of injury is important. In addition to palpation and positive lateral femur X-ray, further imaging studies with proximal femur CT or MRI should be conducted to confirm the presence of femoral neck fracture, if necessary. In some severely displaced fractures, fracture reduction is difficult and requires an open reduction. Based our case reports, in children with femoral neck and ipsilateral femoral shaft fractures, we recommend the use of cannulated screws to reduce the femoral neck, elastic intramedullary closure, reduction and fixation of femoral shaft fractures.

## Author contributions

**Conceptualization:** Shuming Huang, Hailin Xing.

**Data curation:** Shuming Huang, Quanzhou Wu, Fang Ye, Jifei Ye.

**Investigation:** Hailin Xing, Chong Wang.

**Methodology:** Quanzhou Wu.

**Project administration:** Hailin Xing.

**Resources:** Quanzhou Wu, Shuhua Lan.

**Supervision:** Quanzhou Wu, Fang Ye.

**Validation:** Quanzhou Wu, Chong Wang.

**Visualization:** Jifei Ye.

**Writing – original draft:** Hailin Xing.

**Writing – review & editing:** Shuming Huang.

## Abbreviations

Abbreviations: 3D = three dimensional, CRIF = closed reduction and internal fixation, CT = computed tomography, ORIF = open reduction and internal fixation.
